# The effect of testing can increase or decrease misinformation susceptibility depending on the retention interval

**DOI:** 10.1186/s41235-017-0081-4

**Published:** 2017-11-22

**Authors:** Ayanna K. Thomas, Leamarie T. Gordon, Paul M. Cernasov, John B. Bulevich

**Affiliations:** 1 Psychology Department, Tufts University, 490 Boston Avenue, Medford, MA 02155 USA; 2 Assumption College, Worcester, MA 01609 USA; 3 Stockton University, Galloway, NJ 08205 USA

**Keywords:** Misinformation, Retrieval enhanced suggestibility, Repeated testing, Attention allocation

## Abstract

Research has consistently demonstrated that testing prior to the presentation of misleading post-event information, within the context of a standard eyewitness misinformation paradigm, results in an increase in the misinformation effect. The present study investigated whether changes in misinformation susceptibility in the context of interim testing are affected by retention interval differences between misinformation presentation and final testing. Further, this study tested possible divergences in original and post-event learning between conditions where elaboration in processing of critical details was encouraged either indirectly, via interim testing, or directly, by visually emphasizing critical details. In two experiments, we compared three groups of participants. All participants were exposed to an event, presented with misleading post-event misinformation, and then given a final test on the original event. One group was given an interim test between the original event and the post-event synopsis. A second was presented with a post-event synopsis in which critical details were visually emphasized. A third group served as a baseline comparison group for which synopsis processing was not manipulated. All experimental phases occurred in a single session in Experiment 1. A 48-hour retention interval was inserted between the post-event synopsis and final test in Experiment 2. In Experiment 1, we found that interim testing and emphasizing critical details increased misinformation susceptibility as compared to that found in the standard misinformation group. In Experiment 2, misinformation susceptibility was reduced in the interim testing group. These results suggest that interim testing and emphasizing critical details influence the rate of original detail forgetting. At a longer retention interval, the benefits of testing in learning emerged.

## Significance

In today’s society we are inundated with misinformation. Misinformation is presented to us through social media, through peer contact, and in some instances, from presumably reliable sources. In these situations, misinformation may alter our original memories, especially if that misinformation is somehow emphasized. The goal of the present research is to examine how emphasizing misinformation may impact memory for an original event within the context of eyewitness memory. We posit that, depending on the method by which the misinformation is emphasized, that misinformation may either be accepted or rejected. The present study employs the highly reliable misinformation paradigm in which participants are exposed to a complex event, followed by the introduction of misleading post-event information. Critically, we investigate how emphasizing misleading post-event information indirectly, through test-related potentiation, or directly during actual misinformation presentation, will influence memories for the original complex event. This investigation has applied significance because it attempts to ascertain the factors that will exacerbate misinformation acceptance as opposed to rejection. The present results demonstrate that both interim testing and emphasizing critical details will result in an increase in reporting of those details. However, interim testing may also promote a reduction in misinformation susceptibility depending on when the final test is administered. We argue that delaying a final test results in the benefits of the testing effect to emerge, even within the context of the misinformation paradigm.

## Background

Eyewitness memory researchers have long been concerned with factors that influence accurate memory for an originally witnessed event. Towards this end, there has been a substantial amount of research dedicated to understanding the misinformation effect. In a typical misinformation experiment, participants witness an original event. The event usually takes the form of a series of slides or a short video depicting a crime. After some retention interval participants are exposed to misleading post-event information in the form of a narrative or suggestive questions. Following misinformation presentation, memory for the original event is assessed. The typical finding is that exposure to misleading post-event information results in reduced access to original event details and increased reporting of misleading post-event details (Frenda, Nichols, & Loftus 2011).

More recently, research has demonstrated the counterintuitive finding that taking a test prior to receiving the misleading post-event narrative results in an enhanced misinformation effect. Dubbed as retrieval enhanced suggestibility (RES), researchers have demonstrated that preceding cued-recall or recognition testing results in even greater disruption to original event details and greater production of misleading post-event details on a final test of memory (Chan, Thomas, & Bulevich 2009; Chan & LaPaglia 2013; Gordon & Thomas 2014, 2017; Gordon, Thomas, & Bulevich 2015; Thomas, Bulevich, & Chan 2010). In a typical RES study, a cued recall test immediately follows original event presentation and precedes the presentation of the misleading narrative. This condition is generally compared to a standard misinformation group who, instead of taking an interim test, perform some unrelated task prior to the presentation of the post-event synopsis. Research suggests that interim testing in this paradigm may increase accessibility of details presented in the synopsis (Thomas et al. 2010), and may result in test-potentiated learning of post-event details (Gordon & Thomas 2014).

Previous research has provided evidence that interim testing between the original event and post-event synopsis may affect attention and encoding processes employed when processing the narrative. For example, Gordon and Thomas (2014) found that participants who took an interim test spent more time reading sentences in the synopsis that included misleading details than participants who did not take an interim test. This difference in processing time resulted in an increase in errors of commission of suggested misleading details presented in the synopsis (see also Gordon et al. 2015; Gordon & Thomas 2017). Further, when processing time was reduced by requiring participants to simultaneously complete a second task when reading the synopsis, Gordon and Thomas (2017) found that participants who had taken an interim test were no more likely to produce misinformation than participants who had not taken an interim test. The authors argued that the secondary task disrupted the additional processing indirectly engendered by the preceding test.

The pattern of results found within the RES eyewitness paradigm is similar to test-potentiation results found in the verbal learning literature. Researchers have consistently found that testing prior to restudy of a given item facilitates performance on a subsequent test of that item (cf., Izawa 1971; Karpicke 2009) and facilitates the learning of new material (Wissman, Rawson, & Pyc 2011). Gordon and Thomas (2014, 2017) demonstrated that including an immediate test of an originally witnessed event led to better recall of details from the post-event narrative on a modified recall test that encouraged multiple responses, and better recall on a cued-recall test the required responding from only the synopsis, as compared to conditions in which participants did not take an immediate test. Similarly, Pastötter, Schicker, Niedernhuber, and Bäuml (2011) demonstrated that the encoding of information presented after a test was as effective as information presented before the test.

One theory proposes that testing facilitates learning of new material, because it improves encoding of the material. Encoding may be facilitated via the unconscious activation of related information during initial testing (cf., Carpenter, 2011; Chan, McDermott, & Roediger, 2006; Grimaldi & Karpicke, 2012). That is, interim memory retrieval may activate the target and target-related information. That activation may facilitate the incorporation of new information into memory. In addition, testing may change participants’ conscious encoding strategies (e.g., Wissman et al. 2011), leading participants to prioritize rehearsing or reviewing information that is related to previous test questions.

Several studies have linked interim testing with changes in post-test encoding strategies. An early study demonstrated that individuals spent more time reading passages after interim testing (Reynolds & Anderson 1982). More recent research has found that interim testing results in sustained attention during subsequent study and reduces mind-wandering (Szpunar, Khan, & Schacter 2013). It is our view that changes in processing associated with misleading narrative details, as a result of interim testing, influences the accessibility of those details in memory. An increase in accessibility may then have influenced the ease with which misleading narrative details came to mind, biasing responding on a final memory test (cf., Baddeley, 1982; Jacoby, Bishara, Hessels, & Hughes 2007).

Changes in accessibility of misleading post-event information should result in an increase in production of that information on a final test of memory. However, a direct comparison of interim testing, which may indirectly influence the processing of post-test information with a manipulation designed to directly influence the processing of post-test information, has not been examined within a misinformation paradigm. We argue that such an examination has both practical and theoretical implications. Practically speaking, there are a variety of methods employed by criminal investigators, news organizations, and even political operatives to make information more salient. It remains unknown whether such manipulations result in long-term disruption of original memories. The goal of the present study is to test whether the impact of indirect (interim testing) and direct (explicit emphasis) methods to emphasize misinformation will have short and longer term consequences of memory for an original event.

Interim testing was compared to emphasizing details in the context of two experiments. Experiment 1 occurred in one testing session. Experiment 2 included a 48-hour retention interval between synopsis presentation and final testing. We hypothesized that, upon immediate testing, misinformation susceptibility and memory for the original event would appear similar between the interim testing and emphasized detail groups. That is, interim testing and emphasizing details were both predicted to increase misinformation production on an immediate final test. However, when the final test is delayed by 48 hours, we predicted that the influence of misinformation on final test reporting would be diminished in both conditions, allowing the benefits of interim testing of original event memory to emerge.

Research has consistently demonstrated that repeated testing results in better memory performance compared to restudy (for review, see Roediger & Butler 2011). Further, several recent studies suggest that interim testing results in semantic or conceptual organization that promotes robust long-term recall (e.g., Congleton & Rajaram 2012, 2014; Roediger & Karpicke 2006a, b; Zaromb & Roediger 2010). Finally, research suggests that, when temporary accessibility dissipates, original responses may regain their dominance (Lustig, Konkel, & Jacoby 2004). Therefore, we expected that interim testing would result in better memory performance for the original event and reduced misleading errors of commission when final testing occurred after 48 hours.

In the present study, we compared the misinformation effect across three groups, a standard Misinformation group, an Interim Testing group, and an Emphasized Details group. The latter groups were used in order to examine how different forms of elaboration encouragement (indirect vs. direct) would impact memory for the original event when final testing occurred immediately or after a 48-hour retention interval. We predicted that both elaboration groups would demonstrate a greater misinformation effect as compared to the standard group when testing immediately followed presentation of misinformation. That is, both groups of participants would be more likely to produce misinformation on the final test of memory, and less likely to produce original event details, because direct and indirect encouragement to process synopsis details would increase the temporary accessibility of those details and bias responding (cf., Thomas et al. 2010). However, when final testing was delayed, we predicted participants in the Interim Testing group would demonstrate better memory for the original event than participants in either the Standard Misinformation or Emphasized Details groups.

## Experiment 1

### Methods

#### Design

The experiment design was a 3 (Item type: Consistent, Neutral, Misleading) × 3 (Group: Standard, Interim Test, Emphasized Detailed) mixed design. Item type was manipulated within subjects, while Group was a between-subjects variable.

#### Participants

Experiment 1 included a group of 132 participants recruited from the Human Participant Pool at Tufts University. Sample size for each experiment was calculated using G*Power 3 (Faul, Erdfelder, Lang, & Buchner 2007). Our goal was to determine the appropriate sample size using moderate parameters (power = 0.80, effect size f = 0.30). Participants ranged in age from 18 to 23 years, all spoke English as their primary language, and had not been previously exposed to the experimental material. Participants were randomly assigned to one of three groups, with an equal number of participants in each group.

#### Materials and procedure

The original event was a 42-minute episode of the television show *24* (20th Century Fox Television 2001). Following the informed consent procedure, participants were instructed to watch with the knowledge that a memory test about the episode would later occur. After viewing the video, participants in the Interim Test Group took an immediate cued recall test on 33 details of the video (e.g., Question: What did the terrorist use to knock out the flight attendant? Answer [not provided to participants]: A hypodermic syringe). Questions were presented via E-prime 2.1 software (Version 2.1; Schneider, Eschman, & Zuccolotto 2002) and participants were required to respond to all questions. No corrective feedback was provided. The 33 questions presented on the interim test were directly associated with the 33 critical details presented in the post-event synopsis. Participants in the Standard and Emphasized Details Misinformation groups played Tetris (a computerized falling-rock puzzle game) instead of taking the first test. Testing and game play lasted 12 minutes. All participants then completed a brief demographic questionnaire and a vocabulary test (Salthouse 1993). Participants were given 8 minutes to complete these tasks.

All participants were then visually presented with the post-event synopsis, with the instructions to read at their own pace. The synopsis was presented visually using E-prime 2.1 in sequential segments. Participants were instructed to read each segment and press the spacebar to move forward. Thirteen segments were presented, and each contained between one and three critical details. A total of 33 critical details were presented; 11 sentences contained misleading information (misleading, e.g., The terrorist knocks the flight attendant unconscious with a chloroform rag), 11 contained information consistent with the video (consistent, e.g., The terrorist knocks the flight attendant unconscious with a hypodermic syringe), and 11 served as neutral, control sentences (neutral, e.g., The terrorist knocks the flight attendant unconscious). The misleading information always involved replacing a specific item with a plausible alternative. Misleading, neutral, and consistent sentences were counterbalanced. Each critical detail appeared only once in the narrative and whether the detail was consistent, neutral, or misleading was counterbalanced across participants. Both focal and non-focal details were manipulated.

**Fig. 1:**
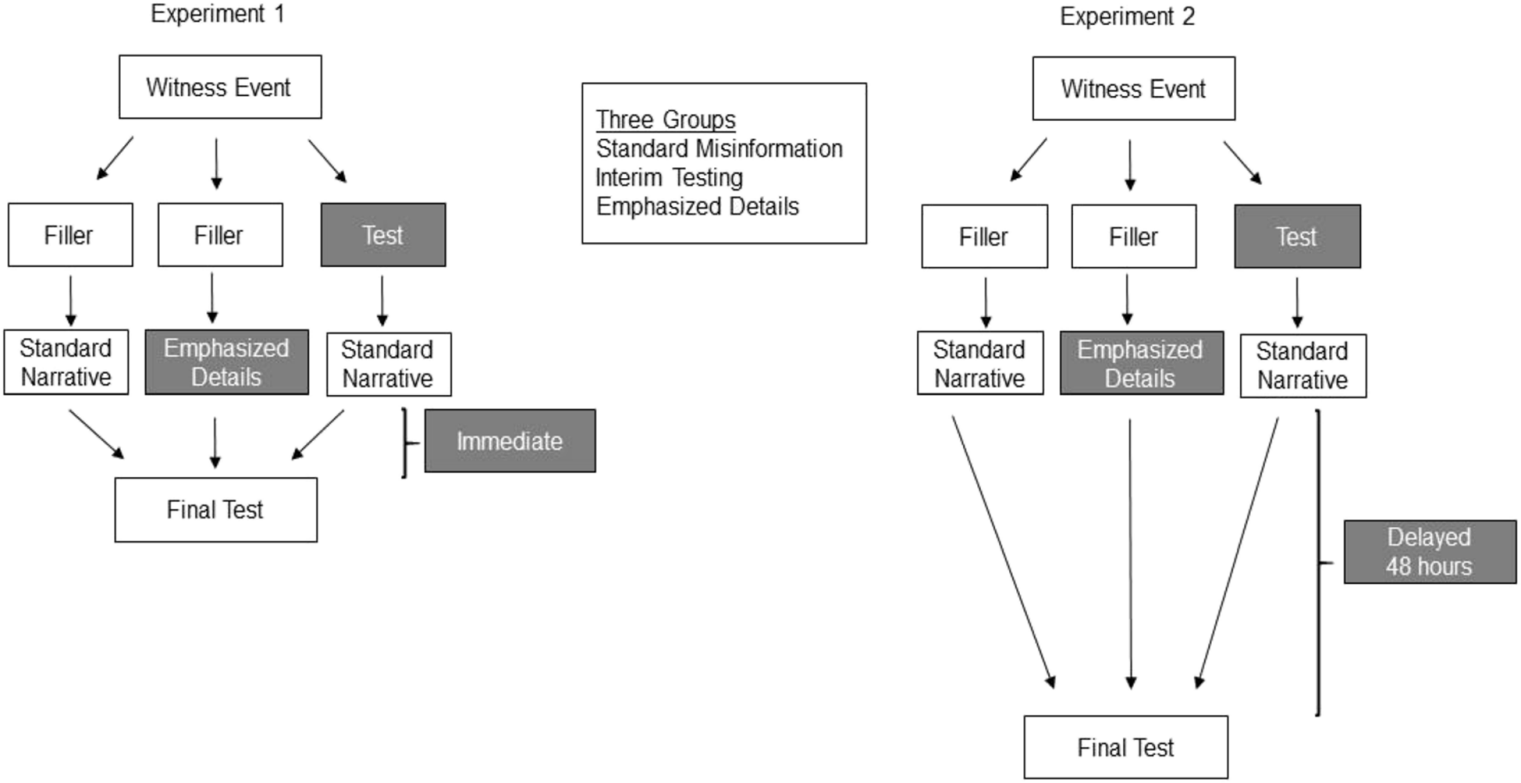
A graphical depiction of the delay schedule and conditions present in Experiments 1 and 2

Participants in the Interim Testing and Standard Misinformation groups received the same narratives. In these groups, the narrative was written in 16-point black Arial font, and presented against a white background. Participants in the Emphasized Details group received the narrative in a similar fashion to the other groups, with one important exception. Sentences containing critical details were presented in red font, and the critical details themselves were underlined. All critical details (consistent, neutral, misleading) were emphasized in this manner. Immediately following the narrative, all participants took a 33-question, forced cued recall test. This test was identical to the one used as the interim test. Participants were instructed to respond with only details from the video, thereby forcing participants to discriminate between the original event and post-event synopsis. Test question order was the same across all groups and followed the narrative structure of the video. Testing was untimed; however, participants could not advance to the next question before responding. A schematic of the procedure can be found in Fig. 1.

### Results

#### Accurate recall on the interim test

All follow-up comparisons used a Bonferroni correction unless otherwise stated. Accurate recall on the interim and final tests was calculated by dividing the total number of trials in which participants produced correct video details by the total number of trials for that given item type. On the interim test, 0.55 of participants’ responses were accurate and 0.05 consisted of spontaneous misinformation production.

#### Accurate recall on the final test

**Fig. 2:**
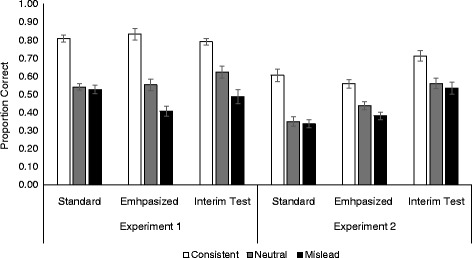
Comparison of accurate detail recall between Experiment 1 and Experiment 2 (means and standard errors plotted)

A 3 (Item type: Consistent, Neutral, Misleading) × 3 (Group: Standard, Interim Testing, Emphasized Details) ANOVA on average final test accuracy found a main effect of item type, *F*(2, 258) = 148.72, *P* < 0.001, ${\eta}_p^2=0.53$. As illustrated in Fig. 2, consistent trials (*M* = 0.81) resulted in significantly greater accuracy as compared to neutral trials (*M* = 0.57, *t*(131) = 11.98, *P* < 0.01, *d* = 1.42). In addition, participants were more accurate on neutral trials compared to misleading trials (*M* = 0.47, *t*(131) = 4.86, *P* < 0.01, *d* = 0.49). We also found an interaction between item type and group (*F*(4, 258) = 4.17, *P* < 0.005, ${\eta}_p^2=0.06$). This interaction was driven by the differences between performance on neutral trials and misleading trials across the three groups. As Fig. 2 illustrates, this difference was small in the Standard Misinformation group, and non-significant when examined using a Bonferroni corrected *t*-test (*t*(43) = 0.40, *P* = 0.70). However, participants in the Emphasized Details group (*t*(43) = 5.31, *P* < 0.001, *d* = 0.69) and participants in the Interim Test group (*t*(43) = 3.51, *P* < 0.001, *d* = 0.58) were significantly less accurate on misleading trials as compared to neutral trials. No other comparisons on final test accuracy were significant.

#### Misleading errors of commission on the final test

**Fig. 3:**
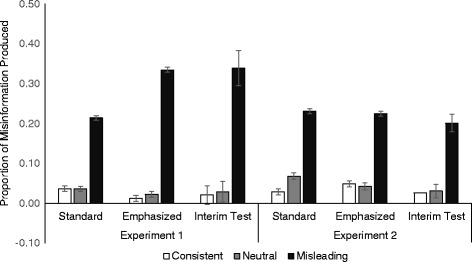
Comparison of misleading detail recall between Experiment 1 and Experiment 2 (means and standard errors plotted)

A 3 (Item type: Consistent, Neutral, Misleading) × 3 (Group: Standard, Interim Testing, Emphasized Details) ANOVA on average misleading errors of commission found a main effect of item type (*F*(2, 258) = 189.12, *P* < 0.001, ${\eta}_p^2=0.59$). As expected, misleading errors of commission were more likely to occur after the presentation of misleading details in the synopsis than spontaneously on consistent or neutral trials. We also found an interaction between item type and group (*F*(4, 258) = 5.27, *P* < 0.005, ${\eta}_p^2=0.08$). Consistent with previous RES literature, participants in the Interim Testing group (*M* = 0.34) were more likely to produce misleading errors of commission on the final test than participants in the Standard misinformation group (*M* = 0.23, *t*(86) = 2.26, *P* < 0.05, *d* = 0.66). Participants in the Emphasized Details group (*M* = 0.33) were also significantly more likely to produce misleading details incorrectly than those in the Standard misinformation group (*t*(86) = 3.25, *P* < 0.005, *d* = 0.49). The difference in mean misleading errors of production between the Interim Test and Emphasized Details group did not reach statistical significance (*t* < 1). These data are presented in Fig. 3.

### Discussion

Experiment 1 demonstrated that misinformation susceptibility was similar for participants in the Interim Test and Emphasized Details groups. That is, participants in these groups demonstrated a greater difference in accuracy between neutral and misleading trials than participants in the Standard misinformation group. Further, these participants were more likely to produce misleading errors of commission on a final test as compared to participants in the Standard misinformation group. Consistent with previous research, these data would suggest that interim testing results in changes to how the post-test narrative is processed. Behaviorally, the increase in misinformation susceptibility was similar to what was demonstrated by highlighting critical details in the present research. Greater susceptibility to misinformation in the context of interim testing and emphasizing details suggests that both procedures may serve to increase accessibility of synopsis details, and that accessibility may influence misinformation error production on the final test. Thus, both interim testing and emphasizing details may result in an ironic effect, boosting suggestibility. Although the findings of the present experiment align with previous research, it remains unclear why interim testing in this eyewitness paradigm does not result in better learning of previously tested information. We hypothesized that such benefits may only emerge when final testing is delayed, because misleading information will no longer exert influence on memory.

## Experiment 2

Experiment 2 explored whether the benefits of interim testing on final memory performance would be more apparent if final testing was delayed. Research has consistently demonstrated that testing effects are more likely to occur when final assessment is delayed. Experiment 2 also had a secondary goal of examining the factors that may dissociate final test performance between the Interim Testing and Emphasized Details groups. Research has consistently demonstrated that testing produces better learning as compared to elaborative encoding (Karpicke & Blunt 2011; Karpicke & Smith 2012). We suggest that the value of interim testing in an eyewitness paradigm may be demonstrated when final testing is delayed and participants are required to rely on more conscious recollective processes to complete the final test. That is, the forgetting that occurs over the 48-hour period should encourage a more effortful search strategy. However, only participants who learned information through interim testing will be able to capitalize on this more effortful search.

### Methods

#### Design

The experiment design was a 3 (Item type: Consistent, Neutral, Misleading) × 3 (Group: Standard, Interim Test, Emphasized Detailed) mixed design. Item type was manipulated within subjects, while Group was a between-subjects variable.

#### Participants

Experiment 2 included a new group of 132 participants recruited from the Human Participant Pool at Tufts University. Sample size for each experiment was calculated using G*Power 3 (Faul et al. 2007).

#### Materials and procedure

We used the same materials in Experiment 2 as used in Experiment 1. After the informed consent procedure, participants viewed the 42 minute video. Participants in the Interim Testing group then took a 33 question cued recall test. Participants in the Standard Misinformation and Emphasized Details group engaged in a filler task. All participants then completed a brief demographic questionnaire, and a vocabulary test (Salthouse 1993). All participants were then visually presented with the post-event synopsis, with the instructions to read at their own pace. The same procedures for narrative presentation used in Experiment 1, were again used in Experiment 2. After the narrative, participants were thanked for their time, and reminded to return to the laboratory 48 hours later. Upon returning for the second session, all participants took a 33 question forced cued recall test. Participants were instructed to respond with only details from the video, thereby forcing participants to discriminate between the original event and post-event synopsis. Testing was untimed; however, participants could not advance to the next question before responding.

### Results

#### Accurate recall on the intervening test

On the intervening test, 0.55 of participants’ responses were accurate and 0.03 of responses were misinformed.

#### Accurate recall on the final test

A 3 (Item type: Consistent, Neutral, Misleading) × 3 (Group: Traditional, Interim Test, Emphasized Details) ANOVA on average correct responding found a main effect of item type (*F*(2, 258) = 82.31, *P* < 0.001, ${\eta}_p^2=0.39$). As illustrated in Fig. 2, consistent trials (*M* = 0.63) resulted in significantly greater accuracy as compared to neutral trials (*M* = 0.45, *t*(131) = 11.26, *P* < 0.01, *d* = 0.87). In addition, participants were marginally more accurate on neutral trials compared to misleading trials (*M* = 0.42, *t*(131) = 1.71, *P* = 0.08). We also found an interaction between item type and group (*F*(4, 258) = 2.95, *P* < 0.05, ${\eta}_p^2=0.04$).

We hypothesized that the positive influence on interim testing on memory performance would emerge in the context of the longer retention interval. As such, we examined this interaction within the context of two subsequent 3 (Item type: Consistent, Neutral, Misleading) × 2 (Group) ANOVAs in which the Interim Testing group was compared to each of the other groups. When Interim Testing was compared with the Standard Misinformation group, we found main effects of item type (*F*(1, 172) = 39.15, *P* < 0.001, ${\eta}_p^2=0.31$) and of group (*F*(1, 86) = 27.18, *P* < 0.001, ${\eta}_p^2=0.25$). On average, participants in the Interim Testing group (*M* = 0.60) demonstrated better final memory test accuracy than participants in the Standard group (*M* = 0.46). The interaction between item type and group was not significant. When the Interim Testing group was compared to the Emphasized Details group, we found a main effect of item type (*F*(1, 172) = 62.58, *P* < 0.001, ${\eta}_p^2=0.42$). We also found an interaction between group and item type (*F*(2, 172) = 5.04, *P* < 0.01, ${\eta}_p^2=0.06$). Although interim testing led to better performance across all items, the difference between the two groups was largest for neutral and misleading trials (consistent: *t*(86) = 2.58, *P* = 0.01 (ns after Bonferroni correction); neutral: *t*(86) = 6.67, *P* < 0.001, *d* = 1.44; misleading: *t*(86) = 4.43, *P* < 0.001, *d* = 0.90).

#### Misleading errors of commission on the final test

A 3 (Item type: Consistent, Neutral, Misleading) × 3 (Group: Traditional, Interim Testing, Details Emphasized) ANOVA on average misleading errors of commission found a main effect of item type (*F*(2, 258) = 167.67, *P* < 0.001, ${\eta}_p^2=0.56$). As expected, misleading errors of commission were more likely to occur after the presentation of misleading details in the synopsis than spontaneously on consistent or neutral trials. No other effects were significant.

#### Additional analyses

Because the only difference between Experiments 1 and 2 was the retention interval that preceded the final test, we also compared misinformation errors of commission on misleading trials (where that information was actually presented) across the two experiments. We found a significant interaction between Group and Experiment (*F*(2, 264) = 2.95, *P* < 0.05, ${\eta}_p^2=0.02$). Retention interval similarly impacted error production for participants in both the Interim Testing and Emphasized Details group. That is, these production errors were higher than those demonstrated by the Standard group in Experiment 1, and dropped to the level demonstrated by the Standard group in Experiment 2. Similarly, when we examined final test accuracy collapsed across item type, we found a significant interaction between Group and Experiment (*F*(2, 264) = 8.59, *P* < 0.001, ${\eta}_p^2=0.06$). However, in the case of accuracy, the interaction was driven by the stable performance across experiments demonstrated by the Interim Testing group (*M*_exp1_ = 0.63; *M*_exp2_ = 0.60). Participants in both the Standard Misinformation and Emphasized Details group demonstrated a drop in final test accuracy between Experiments 1 and 2. These comparisons are presented in Figs. 2 and 3.

## General discussion

The present study demonstrated that both interim testing and visually emphasizing critical details in the post-event narrative influenced misinformation susceptibility. However, and most importantly, the present study suggests that the underlying process by which interim testing and emphasizing details operate are quite different. In Experiment 1, when final testing occurred immediately after the presentation of the post-event synopsis, participants in the Interim Testing and Emphasized Details groups were significantly less likely to correctly remember original event details on misleading trials, and significantly more likely to produce misleading details than participants who were exposed to the synopsis in the absence of testing or emphasis. On the surface, these data would suggest that the encoding operations facilitated by testing might be similar to that instantiated by emphasizing details. In both situations, attention to detail in the narrative may be influenced indirectly by the preceding test or more directly by visually emphasizing those details in the narrative.

Gordon and Thomas (2014, 2017) presented evidence to suggest that interim testing does influence attention and encoding processes of subsequent information in a misinformation paradigm. Specifically, we found that participants spent more time reading sentences in the synopsis that included critical details if a test preceded the synopsis. Further, Gordon et al. (2015) found that both test questions and responses guided subsequent changes in reading time associated with synopsis details. When participants encountered synopsis details that contradicted interim test responses, participants spent more time on those details than when test responses and synopsis details agreed. Consistent with these findings, the present study demonstrated that interim testing influenced how the post-test synopsis was processed and encoded.

We argue that increased misinformation production is directly related to the attention directed to those details, and are akin to what Pastötter and Bäuml (2014) characterize as a forward effect of testing. Pastötter et al. (2011) presented compelling evidence that interim testing may reset encoding processes, resulting in effective learning of post-test information. The forward effects of testing, as discussed thus far in the literature, do not directly necessitate testing. Rather, such effects have been characterized as a ‘reset’ of encoding processes (Pastötter et al. 2011) or a change in encoding strategies (Wissman et al. 2011). As such, the present study examined whether interim testing and other methods employed to affect encoding strategies would have the same downstream consequences for misinformation susceptibility. We found that visually emphasizing critical details resulted in the same pattern of misinformation utilization as interim testing in Experiment 1. However, the impact of emphasizing details and interim testing diverged when memory was assessed at longer retention interval. The pattern of results associated with emphasizing details is consistent with those reported by Eslick, Fazio, and Marsh (2011), who found that highlighting correct and misleading general knowledge details in fiction stories resulted in an increased likelihood of using those incorrect details to answer questions on a subsequent general knowledge test. Although highlighting increased suggestibility, Eslick et al. (2011) also found that highlighted details were more memorable than non-highlighted details; however, participants did not remember the source of those details.

When a 48-hour delay was introduced prior to the final memory test, neither the Interim Testing group nor the Emphasized Details group demonstrated a greater likelihood of producing misinformation on the final test when compared to the Standard Misinformation group.

However, the Interim Testing group demonstrated a greater likelihood of reporting correct details as compared to the Emphasized Details group. These comparisons highlight the differences in learning that underlie interim testing and highlighting or emphasizing details. Both conditions result in access to information for which attention is drawn via test-potentiation or highlighting. However, testing also confers a benefit to information that was initially tested. At a longer retention interval, the influence of attended to misleading details dissipates, allowing for the benefits of testing to emerge. This high commission error rate declined to rates comparable to those found in the Standard Misinformation group when testing was delayed. These results suggest that participants who took an interim test demonstrated a standard testing effect and potentially better contextual discrimination between the two learning episodes (cf., Roediger & Karpicke 2006a, b; Whiffen & Karpicke 2017). Interim testing may have also improved discrimination between the original event and post-synopsis by reducing the search set size (cf., Bäuml & Kliegl 2013).

Notably, the pattern of results found in Experiment 2 contrast with other RES findings where high rates of suggestibility and memory impairment remain, even when final testing is delayed (e.g., Chan & Langley 2011). Chan and Langley (2011) examined interim testing in the context of the misinformation paradigm where final testing was delayed by a week; when final testing was delayed by a week, participants were still more likely to incorrectly report misleading details on the final test of memory if they had taken an interim test. It is puzzling that the value of interim testing for improving monitoring between the original event narrative and post-event narrative did not emerge at a longer retention interval. However, the nature of narrative presentation may have contributed to this pattern of results. Chan and Langley (2011) used an audio narrative, which, by design, conforms to experimenter-paced presentation. We suggest that an experimenter-paced presentation may reduce post-event detail elaboration. Participants may not be able to internally elaborate upon information presented after the test as effectively when presented with experimenter-paced post-event synopsis as compared to self-paced post-event synopsis. When the processing of the narrative is self-paced (Gordon et al. 2015), participants spend significantly more time processing critical details, which may not have been possible with an experimenter-paced narrative. Further, when additional processing of critical details was disrupted by a secondary task, the RES effect was eliminated (Gordon & Thomas 2017). While the present research did not examine the self-paced versus experimenter-paced processing of the post-event information, these results suggest that it may be an important factor in understanding the RES effect.

As opposed to disrupting memory for the original event, as proposed by Chan and LaPaglia (2013), the results of the present study indicate that interim testing may benefit memory for the original event, and tentatively suggest that interim testing may improve discrimination between original and post-event details. The present findings join a growing body of research that suggests that interim testing may not always result in RES, and may actually be beneficial for eyewitness memory. For example, Thomas et al. (2010) found that a warning about the post-event details, and instructions that encouraged participants to discriminate between original event and post-event details, eliminated RES. Under these instructions, participants who took an interim test demonstrated superior memory for the original event when compared to participants who did not take the intervening test. Further, Gordon and Thomas (2014) demonstrated that interim testing resulted in better memory for original and post-event details when participants were allowed to provide multiple responses to final test questions.

Thomas et al. (2010) argued that interim testing may influence accessibility of post-event details. Enhanced accessibility at final test resulted in biased responding. That is, participants are more likely to respond with the most accessible detail. However, previous research has suggested that one of the benefits of testing is that it creates a shift in context (Criss & Shiffrin 2004; Jang & Huber 2008; Whiffen & Karpicke, 2017). Test-potentiated accessibility may bias responding, but that bias can be corrected by instantiating careful retrieval monitoring processes. In situations where participants are given a warning (Thomas et al. 2010) or the 48 hour retention interval in the present research, a careful search of memory may allow participants to take advantage of the differential contexts. Thus, testing between the original event and post-event synopsis should result in both standard benefits of retrieval practice and test-potentiated learning. RES may result from retrieval monitoring failures.

## Conclusions

Consistent with the growing body of research examining the forward effects of testing, the present study reflects the powerful benefits of testing, and the differences between learning information presented after a test as compared to when that information directly externally emphasized. Testing seems to result in robust learning of information presented before and after the test. Direct emphasis seems to result in more superficial learning that results in biased responding. Further, interim testing seems to have similar beneficial effects on eyewitness memory as has been shown in the context of verbal paired-associate and expository text learning. The implications for eyewitness memory are dramatic. Cognitive psychologists have consistently warned of the perils of post-event information on eyewitness memory, which have only increased with the swift changes in how we access information. Many cognitive psychologists spend their time as expert witnesses in criminal cases expounding on the fragility of eyewitness memory, and the ease with which memory for an event can be corrupted by post-event information. The present research does not deny the fragility of eyewitness memory, and suggests that emphasizing details directly may increase biased responding. However, the present research suggests that interim testing may assist in mitigating the consequences of frequently encountered post-event information provided that appropriate monitoring during retrieval can be emphasized.
